# Preparation and Characterization of Lanthanum-Incorporated Hydroxyapatite Coatings on Titanium Substrates

**DOI:** 10.3390/ijms160921070

**Published:** 2015-09-02

**Authors:** Weiwei Lou, Yiwen Dong, Hualin Zhang, Yifan Jin, Xiaohui Hu, Jianfeng Ma, Jinsong Liu, Gang Wu

**Affiliations:** 1 School and Hospital of Stomatology, Wenzhou Medical University, Wenzhou 325027, China; E-Mails: louweiwei1125@163.com (W.L.); triangle163@163.com (Y.D.); evajin1217@163.com (Y.J.); hxh06060@yahoo.com (X.H.); 2 Department of Prosthetic Dentistry, the First Affiliated Hospital, College of Medicine, Zhejiang University, Hangzhou 310006, China; 3 Department of Prosthetic Dentistry, College of Stomatology, Ningxia Medical University, Yinchuan 750004, China; E-Mail: triangle1988@126.com; 4 Department of Oral Implantology and Prosthetic Dentistry, Academic Centre for Dentistry Amsterdam (ACTA), Research Institute MOVE, VU University and University of Amsterdam, Amsterdam 1081 HV, The Netherlands; E-Mail: g.wu@acta.nl

**Keywords:** hydroxyapatite, lanthanum, sol-gel, coating, degradation

## Abstract

Titanium (Ti) has been widely used in clinical applications for its excellent biocompatibility and mechanical properties. However, the bioinertness of the surface of Ti has motivated researchers to improve the physicochemical and biological properties of the implants through various surface modifications, such as coatings. For this purpose, we prepared a novel bioactive material, a lanthanum-incorporated hydroxyapatite (La-HA) coating, using a dip-coating technique with a La-HA sol along with post-heat treatment. The XRD, FTIR and EDX results presented in this paper confirmed that lanthanum was successfully incorporated into the structure of HA. The La-HA coating was composed of rod-like particles which densely compacted together without microcracks. The results of the interfacial shear strength test indicated that the incorporation of lanthanum increased the bonding strength of the HA coating. The mass loss ratios under acidic conditions (pH = 5.5) suggested that the La-HA coatings have better acid resistance. The cytocompatibility of the La-HA coating was also revealed by the relative activity of alkaline phosphatase, cellular morphology and cell proliferation assay *in vitro*. The present study suggested that La-HA coated on Ti has promising potential for applications in the development of a new type of bioactive coating for metal implants.

## 1. Introduction

Titanium (Ti) and its alloys have been widely used as biomedical devices in clinical applications, particularly in dental implantology [1]. A previous study reported that the success rates of dental implants composed of titanium were 90%–95% in medically healthy patients [2]. However, titanium implant failures still remain [3]. As the population ages, the incidence of implant failure will be high in patients who have severe alveolar bone absorption and/or poor bone quality [4,5]. To enhance the bioactivity of bioinert titanium would be favourable to improving osseointegration of the implants and thus minimizing the risk of implant failures. This may be achieved through surface modifications, and the deposition of hydroxyapatite [Ca_10_(PO_4_)_6_(OH)_2_, HA] coatings on Ti substrates is generally applied. HA has become one of the most widely used biomedical materials because of its similar properties to bone [6,7]. Therefore, the strategies of coating Ti implants with HA coatings may combine the good mechanical properties of Ti with the excellent bioactivity of HA. However, the chemical composition of HA coatings still differs from that of biological apatite in bone. Biological apatite is defined as nonstoichiometric apatite with various substitutions of other metallic ions for calcium ions. The incorporation of some elements, such as silicon and strontium, into the apatite crystal lattice could significantly improve the physicochemical and biological performances of HA [7,8,9,10,11].

Naturally, the effects of rare earth elements (REEs) on human health have been attracting increased attention in orthopaedic fields. REEs, including all lanthanide elements (such as lanthanum, cerium, and praseodymium), can be found in bovine whole blood [12]. In general, La and other REEs are known as Ca-substituting ions in apatite [13]. The incorporation of La^3+^ in HA improves both the physicochemical and biological properties of HA. Serret *et al.*, showed that the incorporation of La into the crystal lattice of hydroxyapatite stabilized its apatite structure [14]. Furthermore, doping La_2_O_3_ into the HA structure improved the tensile and bending strengths of HA [15]. La-containing HA discs exhibited good formation of fibrous tissue around the discs without significant inflammation when they were implanted in the bones of rats [16]. Fernandez *et al.* reported that the substitution of La for Ca in apatite increased the resistance of hard tissues to acid dissolution [17]. More interestingly, Zhang *et al.* found that La^3+^ at a concentration of 1.00 × 10^−^^5^ mol/L significantly inhibited bone resorption [18]. These invaluable properties confer La a promising application potential to improve the performance of dental implants in osteoporotic cases. Although HA coatings have been used to improve the bioactivity of dental implants, the application potential of La-incorporated coating on dental implants has not yet been reported. In this study, we wished to (1) for the first time, develop La-HA coatings on Ti substrates; and (2) investigate their the physic-chemical and biological properties.

## 2. Results and Discussion

### 2.1. XRD Results

**Figure 1:**
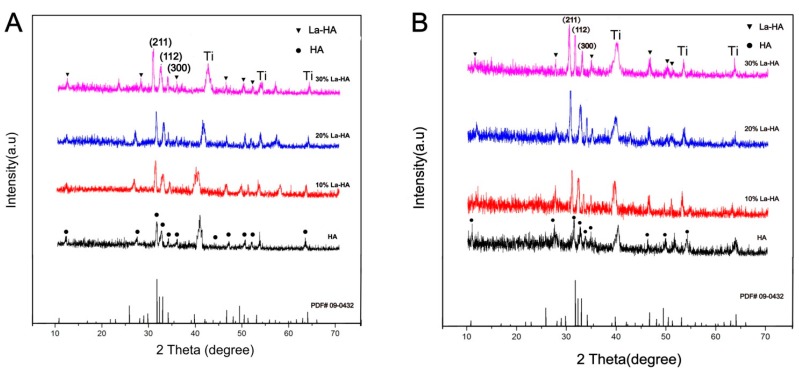
X-ray diffraction (XRD) patterns of HA and La-HA coatings (**A**) without sintering and (**B**) sintered at 600 °C.

Figure 1A presents the XRD patterns of the as-prepared HA and La-HA coatings on Ti substrates together with the standard diffraction data of HA. It is evident that all the coatings give similar XRD patterns. Their peaks are in agreement with the standard data of JCPDS Card No. 09-0432, confirming the formation of HA coatings without any other calcium phosphate phases. Despite the different La contents, no characteristic diffraction peaks of La(NO_3_)_3_ or La_2_O_3_ were recorded in three La-HA coatings. However, downward shifting of these reflections was recorded in La-HA coating. For example, in contrast to the (211) reflection of pure HA at 31.792° (2θ), this peak shifted to 31.542° for 10% La-HA sample, and to 31.082° for 30% La-HA sample. This shifting contributes to the larger lattice space of La-HA due to the partial substitution of La^3+^ with a larger radius (0.1016 nm) for Ca^2+^ with a smaller radius (0.099 nm), confirming that La^3+^ partially substituted Ca^2+^ in the apatite lattice [19,20,21,22]. In addition, the reflections of La-HA coatings gradually became sharpened as the La content increased, suggesting that the presence of La in the coating led to enhanced crystallinity. The XRD patterns of coating samples sintered at 600 °C are shown in Figure 1B. A higher sintering temperature is associated with higher peak intensity and thus a higher degree of crystallization for both coatings. In Figure 1B, the (211) peaks shifted towards lower diffraction angles as the content of La increased, including the 20% La-HA coating. The peak shifted to 31.452° for the 10% La-HA, 31.112° for 20% La-HA and 30.972° for 30% La-HA. This result indicated that the heat treatment provided higher external energy to introduce La into the HA crystal structure. The XRD results indicated that La was successfully introduced into the apatite lattice. The introduction of La improved the degree of crystallization, and the heat treatment provided a higher external energy to introduce La into the HA crystal structure.

### 2.2. FTIR and EDX Results

**Figure 2:**
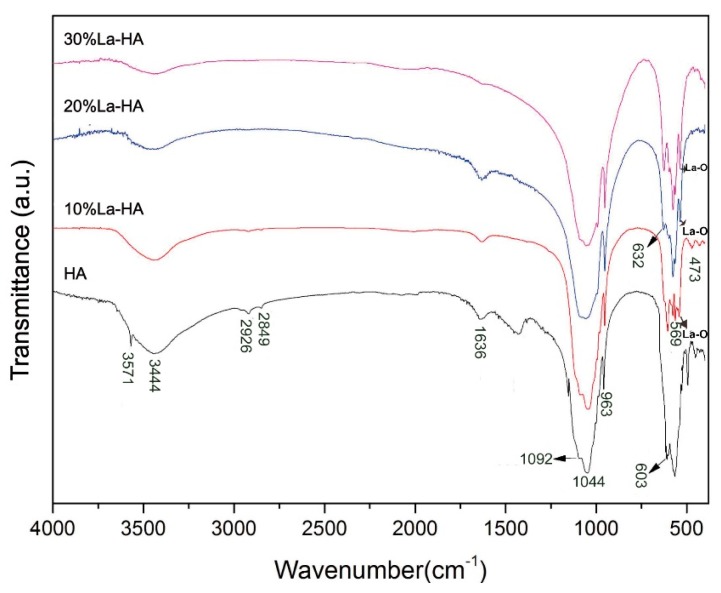
Fourier transform infrared (FTIR) spectra of as-prepared coatings.

The FTIR results of as-prepared coatings (Figure 2) indicated the chemical groups present in the coatings. The spectral bands at 603, 569, 963, 1044 and 1092 cm^−1^, as well as that at 473 cm^−1^, were attributed to PO_4_^3−^ groups in HA [19]. The bands at 3444 and 1636 cm^−1^ corresponded to water absorbed in as-prepared coatings. In general, the structural OH^−^ groups of well-formed HA give characteristic adsorptions at 3571 and 631 cm^−^^1^. However, the adsorption at 3571 cm^−1^ was only detected in the pure HA coating whereas the 632 cm^−^^1^ adsorption was only detected in La-HA coatings, especially with a higher La content. It is plausible that this spectral change was due to the introduction of La in HA. The band between 510 and 525 cm^−1^ was the La-O absorption band [20]. This absorption band became more obvious in La-HA coatings as the content of La increased. However, the absorption bands at 882, 1421 and 1460 cm^−1^ for carbonate groups were invisible in La-HA coatings, which reflected the purity of the sol-gel derived La-HA coatings.

**Figure 3:**
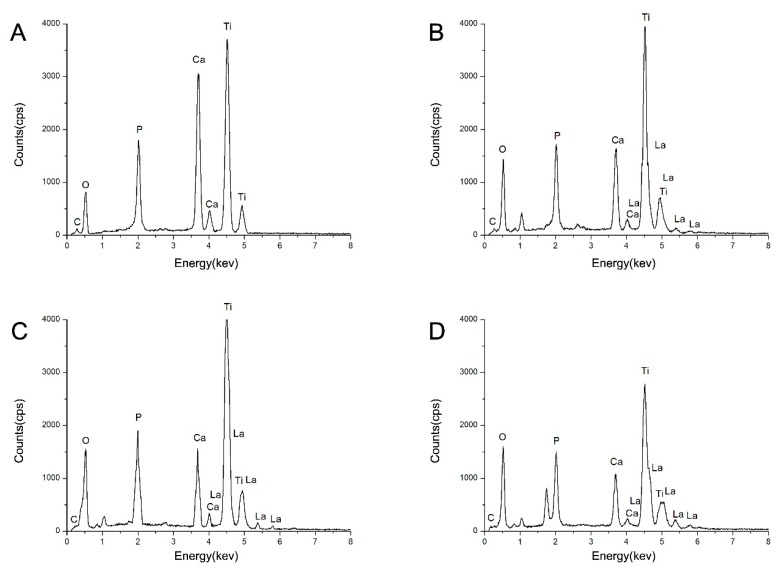
Energy-dispersive X-ray (EDX) spectra of as-prepared coatings. (**A**) HA coating; (**B**) 10% La-HA coating; (**C**) 20% La-HA coating and (**D**) 30% La-HA coating.

Figure 3 presents the EDX spectra, which characterize the chemical elements present in the as-prepared coatings. The characteristic peaks of Ca, P, O, C, and Ti elements were present in all of the four experimental groups. The characteristic peaks of La were detected in the La-HA coatings, and the intensity of the La peaks increased as the content of La increased, as shown in Table 1. According to the XRD, FTIR and EDX results, these important variations were detected in the La-HA coatings as the La content increased—for instance, the shift of the diffraction peaks (211), the spectral change in the characteristic adsorptions of structural OH groups and the increased absorption intensity of the La-O band in the FTIR results, due to the substitution of larger La ions (0.1016 nm) for Ca ions (0.099 nm) [21,22].

**Table 1:** Quantitative EDX analysis of as-prepared coatings (wt %: weight percent, At %: atom percent).

Element	HA		10% La-HA		20% La-HA		30% La-HA	
	wt %	At %	wt %	At %	wt %	At %	wt %	At %
P	8.92	8.26	8.42	7.40	7.14	6.72	7.95	7.93
Ca	21.06	15.11	8.03	5.47	8.53	6.22	6.29	4.86
La	0	0	5.43	1.06	9.81	2.06	25.61	5.70
Ti	40.93	24.47	41.37	23.48	41.84	25.43	26.98	17.39
O	29.08	52.16	36.75	62.58	32.67	59.57	33.16	64.11

### 2.3. Morphology of Coating Surface

**Figure 4:**
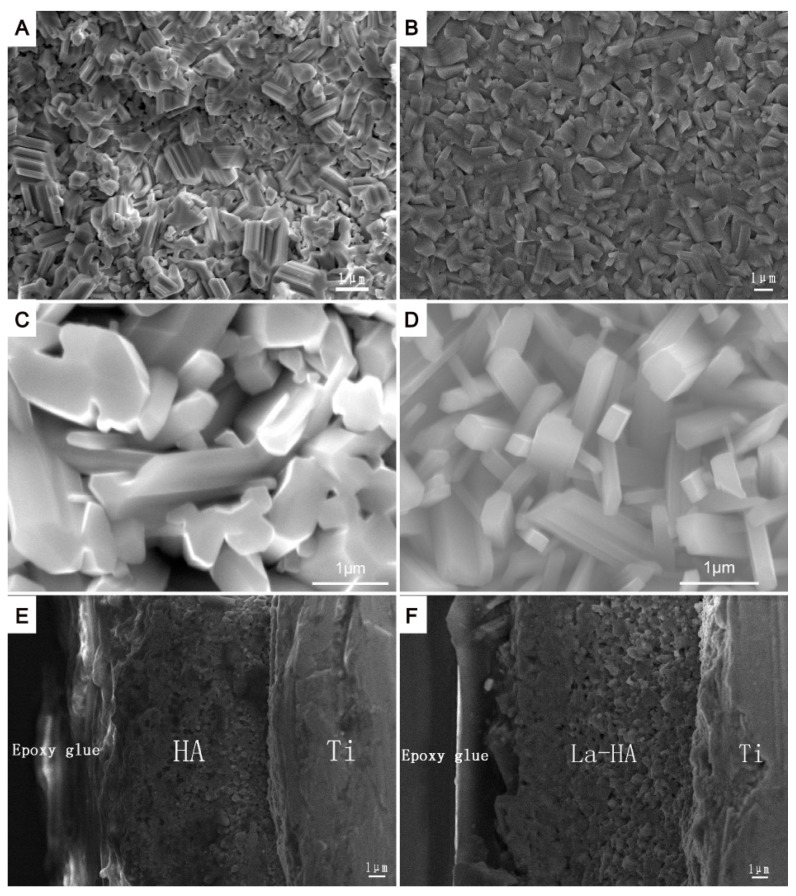
SEM images of two coating systems on Ti substrates without heat treatment. (**A**) HA coating; (**B**) 10% La-HA coating; (**C**) HA coating at high-magnification; (**D**) 10% La-HA coating at high-magnification; (**E**) HA coating with a cross-sectional view; and (**F**) 10% La-HA coating with a cross-sectional view. Scale bar = 1 μm.

SEM micrographs of HA and 10% La-HA coatings without heat treatment are shown in Figure 4. Both coatings were composed of rod-like particles which densely compacted together (Figure 4A,B). The magnified images (Figure 4C,D) further revealed that the particles of the La-HA coating were more uniform than those of the HA coating, and there were no visible micro-cracks on either the La-HA or HA coatings. The diameter of rod-like crystals in La-HA coatings was in the range of 108–500 nm. The La-HA coating had a rough surface microstructure similar to that of the HA coating. The rough surface of HA coatings has been reported to be favourable for cell attachment and proliferation [23,24]. The cross-sectional images (Figure 4E,F) showed the coatings had a homogeneous thickness of approximately 7 μm. In fact, the coating thickness could be easily adjusted by controlling the deposition times during the dip-withdrawal process [25]. The cross-sectional view also confirmed that the coating was densely compacted without visible micro-cracks. Additionally, there was no delamination at the sectioned interface between the coating and Ti, suggesting that the La-HA coatings possessed a very favourable bonding capability.

**Figure 5:**
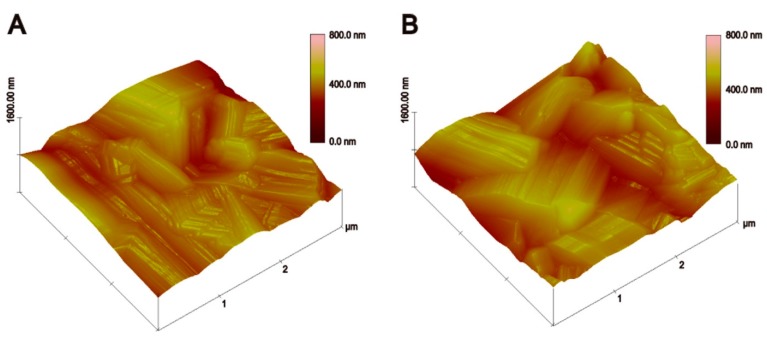
AFM images of the surfaces of (**A**) HA and (**B**) 10% La-HA coatings sintered at 600 °C.

Figure 5 shows the surface topographies of the HA and La-HA coatings as evaluated using AFM. Both coatings were composed of rod-like particles with edges. Within the limits of the AFM observations, the mean surface roughness of the La-HA crystal was 68.17 nm, and the mean surface roughness of the HA coating was 70.43 nm. An appropriately roughened surface has been proven to improve cell adhesion, spreading, proliferation and differentiation [26]. Furthermore, in clinical trials, it has been demonstrated that a roughened implant surface increased the amount of translocated bone particles and thereby led to beneficial osteogenic responses, which is similar to autografts improving peri-implant osteogenesis [27,28,29]. The surface morphologies of the different La-HA coatings and HA coating showed that the introduction of La into the apatite lattice made the coatings more uniform. Considering that the bonding capability is important for clinical use, the cross-sectional view of the La-HA coatings, which was densely compacted without visible micro-cracks, suggested that the La-HA coatings had a favourable bonding capability.

### 2.4. Bonding Strengths of the Coating Layers

**Figure 6:**
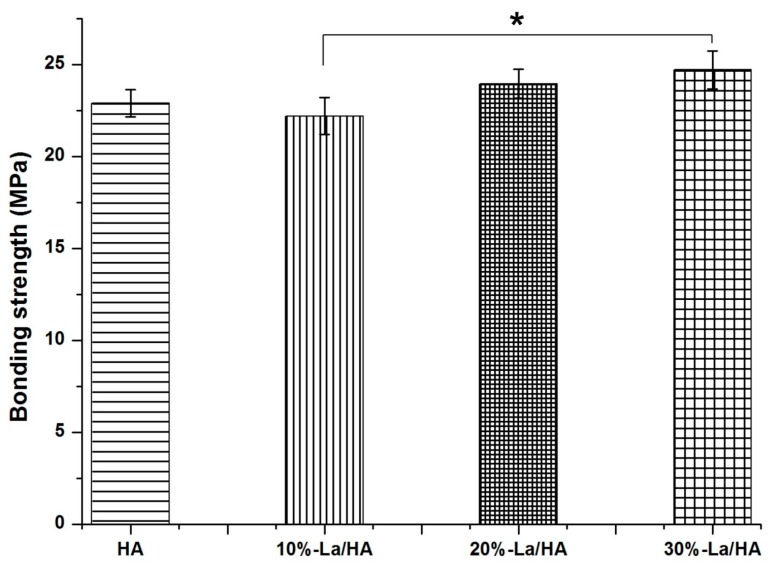
Bonding strengths of coatings on Ti substrates sintered at 600 °C. All data are presented as the mean values together with the standard deviation. *: *p* < 0.05.

Figure 6 shows the bonding strengths of HA and La-HA coatings sintered at 600 °C. The bonding strengths for HA, 10% La-HA, 20% La-HA and 30% La-HA coatings were 22.9 ± 0.7, 22.2 ± 1.0, 24.0 ± 0.8 and 24.7 ± 1.0 MPa, respectively, which were higher than those in previous studies for electrophoretic and sol-gel HA coatings (7~27 MPa) [30,31,32,33]. The La-HA coatings achieved slightly higher mean values at La contents of 20% and 30% compared that for the pure HA coating. In contrast to the pure HA coating, the values for the 10% La-HA coating decreased, but there was no significant difference between them, which indicated that the introduction of La did not decrease the bonding strength of the HA coating. However, there was an obvious significant difference between the 10% and 30% La-HA coatings: the values for the 20% and 30% La-HA coatings were slightly higher than that for the 10% La-HA coating, which revealed that the bonding strength increased as the La content increased. This result may be related to the densely compacted cross-section of the coatings on the Ti substrate with micro-cracks, as shown in Figure 4. The good adhesion strength of the coating with titanium substrate is also crucial for the long-term stability of the implant [34]. Coatings with poor bonding strength may easily detach from the implant, which may trigger diseases and eventually implant loosening [35]. The present results confirmed that the dip-coating technique was successful for depositing La-HA onto titanium substrates with enhanced adhesion strength.

### 2.5. Degradation of Coatings

**Figure 7:**
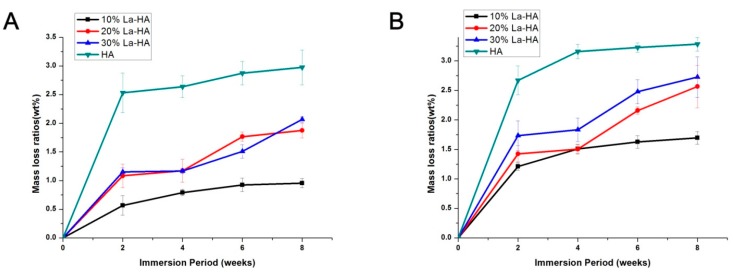
Mass loss ratios of coatings sintered at 600 °C (**A**) at pH = 7.4 and (**B**) pH = 5.5.

Figure 7A,B show the mass loss ratios of coatings under different pH conditions (7.4 or 5.5). Under both pH conditions, the mass loss ratios of the La-HA coatings were significantly lower than that of the pure HA coating throughout the entire experimental period, which confirmed that the introduction of La decreased the degradation rate of the HA coating under both conditions. As shown in Figure 7A (pH = 7.4), after 6 weeks, the degradation rate of HA tended to be more stable than that of La-HA (La content = 30%). This result may due to too much La in the apatite crystal structure, which reduced the stability of the structure. In contrast, the La-HA coating with a La content of 10% showed a stable degradation rate and clearly lower mass loss ratios than all the other coatings within the experimental period. This indicated that the 10% La-HA coating had better degradation resistance and acid resistance. All of the above results suggested that the resistance of HA to degradation was improved by the incorporation of La^3+^, and the La-HA coatings also had better acidic resistance than the pure HA coating, which would lead to longer survival of the coatings on the surface of Ti substrates [36]. Within the limits of our experiment, the 10% La-HA coating exhibited better acid resistance and a lower degradation rate.

HA-based coatings are well known to degrade in bone tissue. It has been demonstrated that an approximately 10~15 μm HA coating may degrade during the first few months after implantation as bone union occurs [37]. The degradation of HA is generally estimated by soaking the materials in an acidic buffer (pH 5.5) and physiological buffer (pH 7.4). The acidic buffer, to some extent, mimics the acidic environment during osteoclastic activity (bone resorption). The degradation of HA coatings is affected by the chemical composition, crystallinity, particle size, Ca/P ratio, preparation conditions, density and extent of ionic substitutions into the apatite lattice [38,39,40]. According to XRD, FTIR SEM and EDX results, the incorporation of lanthanum led to formation of uniform, crack-free La-HA coatings with enhanced crystallity, which contributed to the measured lower degradation rate of the La-HA coating [7,41]. The sustained release of incorporated ions, calcium and phosphate around the implant surface promotes the growth of the bone by increasing local supersaturation [42].

### 2.6. Cell Proliferation Assay

**Figure 8:**
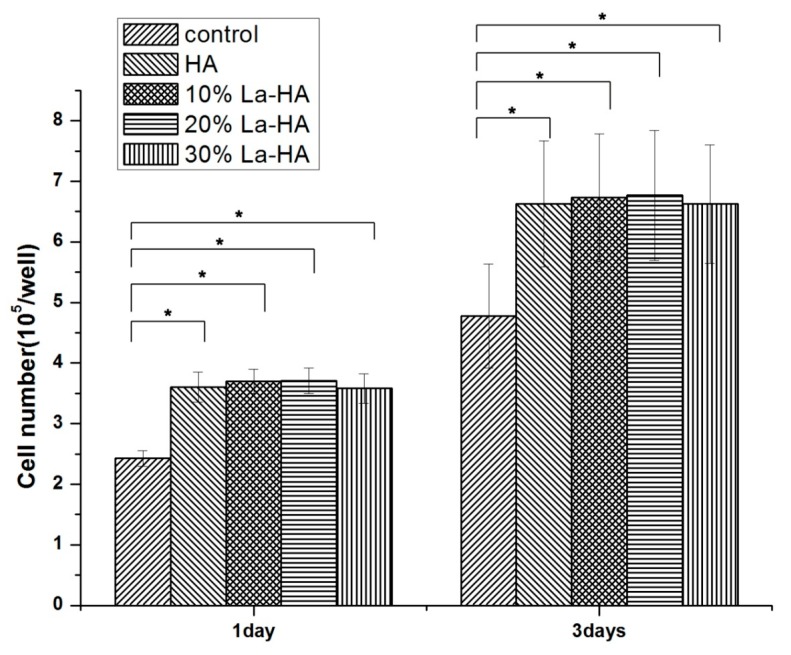
The number of MC3T3-E1 cells on different coatings sintered at 600 °C for 1 and 3 days of culture. All data are presented as the mean values together with the standard deviation. *: *p* < 0.05.

After culturing on the coated titanium substrate for 1 and 3 days, the cells on the coating surface were counted to investigate the cell proliferation, and the results are shown in Figure 8. The cell numbers on the La-HA coatings (10% and 20%) were higher than that on the HA coating, and there was no obvious difference between the values of pure HA and 30% La-HA (*p* > 0.05). The effect of La (10% and 20%) which was incorporated into HA apatite structure to cell proliferation was limited in our study but showed a positive trend. In Liu’s study, with the concentration of 1 × 10^−8^ mol/L, La obviously prompted the proliferation to MC3T3-E1 cells, while with the concentration of 1 × 10^−6^ mol/L, there were no obvious difference [43]. The values in Figure 7 indicated that the degradation of the coatings in first two weeks were high, while after two weeks the degradation of the coatings decreased and remained at a low level for a month. It figured out that the concentration of La which released from the coatings may be close to 1 × 10^−6^ mol/L in the first two weeks, but the concentration of released La decreased after that and remained at low values for a month, which resulted in the values of the cell number. Meantime, there was no obvious cytotoxicity of La-HA coatings with different contents, which indicated that the La-HA coatings were a promising biomaterial for clinical use.

### 2.7. Alkaline Phosphatase (ALP) Activity Assay and Cellular Morphology

**Figure 9:**
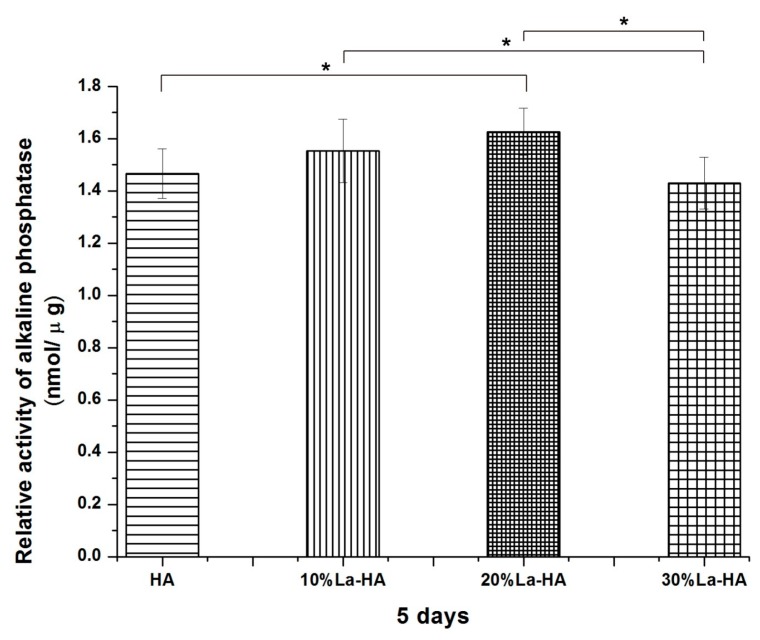
The relative ALP activities of MC3T3-E1 cells cultured on different coatings (sintered at 600 °C) for 5 days. All data are presented as the mean values together with the standard deviation. *: *p* < 0.05.

The ALP activity and total protein content were measured to assess the early differentiation of pre-osteoblasts. Figure 9 shows the relative ALP activity of MC3T3-E1 cells seeded on different coatings for 5 days. The relative ALP activities in the 10% La-HA and 20% La-HA groups were higher than that of the pure HA group, especially in the content of 20%. In contrast, the values for the 30% La-HA was lower than that for pure HA, but the difference was not significant (*p* > 0.05). These results indicated that the La-HA coating slightly promoted the differentiation of osteoblasts when the proper ratio of La was introduced. It has been confirmed that La^3+^ enhances osteoblast differentiation by ERK (extracellular signal-regulated kinase) activation via Pertussis Toxin-Sensitive Gi Protein signalling [27].

**Figure 10:**
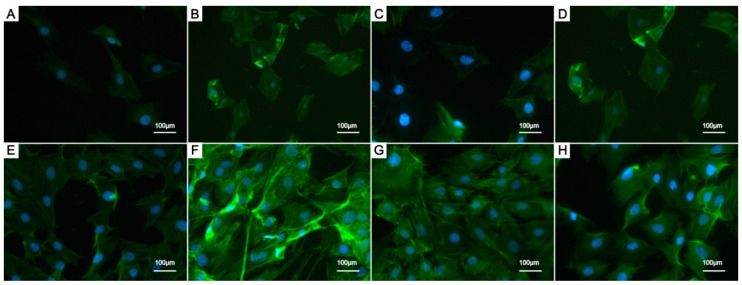
Cell morphologies of MC3T3-E1 cells seeded on HA coating (**A**,**E**); 10% La-HA coating (**B**,**F**); 20% La-HA coating (**C**,**G**); and 30% La-HA coating (**D**,**H**) for 1 and 3 days. All the coatings have been sintered at 600 °C. Scale bar = 100 μm.

Figure 10 shows the morphologies of cells seeded on different coatings. The cells appeared elongated and spread out on all coatings, and the F-actin filaments extended in numerous directions, which revealed the good cytocompatibility of the coatings. The F-actin filaments of cells on the 10% and 20% La-HA coatings (Figure 10F,G) extended in a manner longer and more pronounced than those on the pure HA coating. It has been reported that La has dose-dependent effects on the proliferation and differentiation of MC3T3-E1 cells. When the concentration of La in the cell culture medium was less than 1.0 × 10^−8^ mol/L, the proliferation and differentiation of MC3T3-E1 cells was enhanced, and when at a concentration of 1 × 10^−^^6^ mol/L, La had no effect on the proliferation of the cells. In contrast, the high concentration, greater than 1 × 10^−^^5^ mol/L induced obvious damage to the cells [43]. The La-HA coating was a highly crystallized structure and had a slow degradation rate. Therefore, within our experiment, the proper content of La introduction in a HA coating showed a positive trend toward cell proliferation and dose-dependent promoted osteogenic differentiation.

## 3. Experimental Section

### 3.1. Preparation of the Ti Substrate Samples

Commercially pure Ti substrates (0.1 cm × 1 cm × 3 cm, TA2 of 96% purity, Shenghua, China) were polished sequentially using 180-, 800- and 1000-grit silicate-carbon papers to remove macro-level surface defects and contaminations. The samples were degreased by sonication in acetone, methanol, and ethyl alcohol, followed by rinsing with deionized (DI) water for 10 min and drying at 100 °C for 30 min in an air oven. These cleaned Ti substrates were further treated with a 5 mol/L NaOH aqueous solution at 60 °C for 24 h.

### 3.2. Preparation of La-HA Sols

Preparation of La-HA sols followed Liu’s work with minor modification [19]. Briefly, La-HA sols were synthesized using a series of stoichiometric mixtures composed of 0.01M monocalcium phosphate [Ca(H_2_PO_4_)_2_], 0.02 M calcium hydroxide [Ca(OH)_2_] and certain amounts of lanthanum nitrate [La(NO_3_)_3_]. A wet chemical method at room temperature was applied, and the pH was kept constant at 9.8 during precipitation by adding ammonium hydroxide (NH_3_·H_2_O, AR) with a pH-stat automatic titrator (HI84429, Hanna, Italy). During the 5-day wet chemical process, ultrasonic vibration was applied adequately to keep the stability of the La-HA sols without sedimentation formation. In this study, three groups of La-HA sols were prepared with La content set at 10%, 20% and 30% according to the molar ratio of La/(Ca + La). As a control group, the pure HA was fabricated with a similar method without introducing lanthanum nitrate. All chemicals were analytical reagents. The as-synthesized La-HA sols and HA control sol were kept at −40 °C for further use.

### 3.3. Preparation of La-HA Coatings

La-HA or HA was deposited on the Ti substrates by a dip coating technique using the as-synthesized sols [44]. The sol-coated substrates were then dried in an air oven for 30 min at 150 °C. Totally, five cycles of dip coating and oven drying were applied to obtain coatings. Four groups of coatings were prepared, and designated as HA, 10% La-HA, 20% La-HA and 30% La-HA coatings. For post heat treatment of coatings, the as-prepared coatings were further sintered at 600 °C for 2 h followed by furnace cooling to room temperature. The heating rate was 5 °C/min.

### 3.4. Characterization of La-HA Coatings

The chemical species of coatings were determined by using FT-IR absorption spectroscopy (FTIR; Equinox 55, Bruker Co., Ettlingen, Germany) with a KBr pellet method. The spectrum was collected in the wavenumber range of 4000–400 cm^−1^ with a scanning resolution of 2 cm^−1^. The crystalline phase of coatings was analyzed by X-ray diffraction (XRD, D8 Focus, Bruker Co.) with the working parameters of 35 kV and 30 mA. The data were recorded in the 2θ range of 10°–70° at a scanning rate of 0.06°/s. Surface and cross-sectional morphologies of the coatings were observed by Field-emission scanning electron microscopy (Nova NanoSEM200, FEI Co., Houston, TX, USA) coupled with Energy dispersive X-ray (EDX) spectroscopy. The surface roughness of coatings was evaluated by atomic force microscope (Veeco MultiMode, Nanoscope IIIa controller, Veeco Co., Somerset, NJ, USA).

### 3.5. Bonding Strengths of Coatings

**Scheme 1:**
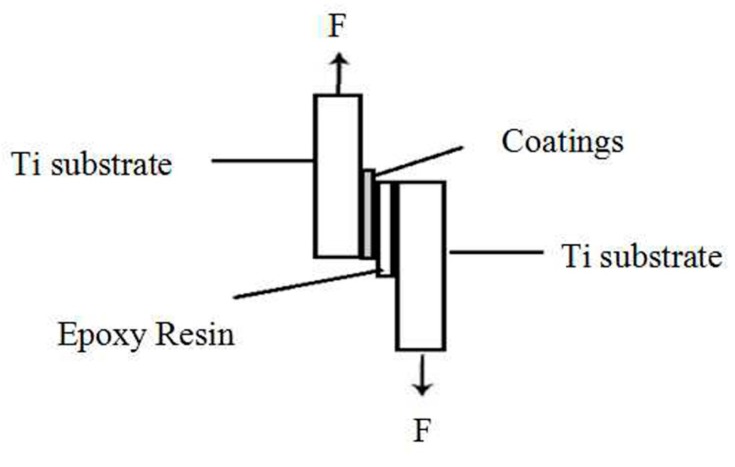
Schematic diagram of the interfacial shear strength test for coatings.

The bonding strengths of the different coatings were evaluated by using a interfacial shear strength method with epoxy glue (pull-out strength of ~60 MPa) [30,44]. The procedure was performed using a universal testing machine (NDN-100, Reger, China) at a rate of 1 mm/min. The coating sample was attached to an uncoated Ti substrate with epoxy glue cured at a temperature of 160 °C for 3 h. The data were presented as the mean ± standard deviation (SD) with six samples *per* group. A schematic diagram of interfacial shear strength test is shown in Scheme 1.

### 3.6. Degradation Evaluation in Vitro

To evaluate the degradation rates of coatings, we calculated the mass loss ratio (*m*_0_) for each sample immersed for weeks according to the following equation: *m*_0_ = 100% (*m*_2_ − *m*_1_ − *m*_3_)/(*m*_2_ − *m*_3_). In this equation, m_2_ denotes the mass of coatings before immersion, m_1_ denotes the mass of coatings after immersion, and m_3_ denotes the mass of Ti substrates before being coated. The tests were performed in two PBS solutions with different pH (pH = 7.4 or pH = 5.5) for 8 weeks at 37 °C. The immersion solution was changed every 2 days. After different incubation periods (2, 4, 6, and 8 weeks), the coatings were removed and weighed after dried for 5 h in a vacuum oven. Five samples *per* group were tested.

### 3.7. Cell Culture

MC3T3-E1 osteoblasts (ATCC; Chinese Academy of since, Shanghai, China) were cultured in a-Minimum Essential Medium (a-MEM) containing 10% foetal bovine serum (FBS) (Gibco, Invitrogen, Grand Island, NY, USA), 10 μg/mL penicillin, 10 μg/mL streptomycin, and 50 μg/mL fungizone. The medium was changed every 3 days. MC3T3-E1 osteoblasts (passage 8–14) were harvested using 0.25% trypsin and 0.1% EDTA, seeded onto HA- or La-HA-coated Ti discs (φ = 1.77 cm) at 1 × 10^4^ cells/cm^2^, and placed in a Petri dish containing α-MEM with 10% FBS.

### 3.8. Cell Proliferation Assay

The cell proliferation of MC3T3-E1 cells was calculated as follows. The cell number of each group was determined using the alamarBlue cell viability reagent (Invitrogen Corporation, Carlsbad, CA, USA) after being seeded on different coatings for 1 and 3 days. A fluorescence spectrometer (SpectraMax M5 Molecular Devices, Sunnyvale, CA, USA) at EX 540 nm/EM 590 nm was used to measure the fluorescence intensity [45]. Six samples per group are used and the experiments are performed in triplicate.

### 3.9. Alkaline Phosphatase (ALP) Activity Assay

The ALP activity and total protein content were measured after cells were seeded on different coatings for 5 days to estimate the early differentiation of osteoblasts. We used a LabAssay™ ALP colorimetric assay kit (Wako Pure Chemicals, Osaka, Japan) to determine the ALP activity in the cell lysate (Sigma-Aldrich, St. Louis, MO, USA). The total protein content was measured at 570 nm using a commercial BCA Protein Assay kit (Beyotime, Beijing, China). The values were expressed as nmol p-NP/ug total protein/hour [45]. Six samples per group are used and the experiments are performed in triplicate.

### 3.10. Cellular Morphology Imaging

Cellular morphology was observed by actin and nucleus staining. After culturing for 1 and 3 days, samples were removed and rinsed three times with PBS solutions at room temperature. Subsequently, the samples were fixed using a 4% paraformaldehyde solution with Tri-ton X-100 for 5 min. Then, the cell nucleus was stained by DAPI (Aladdin, Shanghai, China) for 5 min and washed twice with PBS. F-actin protein was stained by phalloidin—FITC (Aladdin) overnight at 4 °C, and then it was washed twice with PBS solution. The cellular morphology was imaged using a fluorescence microscope.

### 3.11. Statistical Analysis

At least 5 samples per group were used and the experiments were performed in triplicate, and the results were reported as the means ± standard deviations (SD). Statistical analysis was performed by Student’s *t*-test and one-way analysis of variance (ANOVA). Difference at *p* < 0.05 was considered to be significant.

## 4. Conclusions

In this study, La-HA coatings were successfully deposited onto Ti substrates by using a dip-coating technique with sols. To the best of our knowledge, this is the first report to develop La-HA coatings with the aim of improving the physicochemical and biological properties of titanium substrates. XRD, FTIR and EDX analyses confirmed the incorporation of La into the structure of HA. The post-heat treatment facilitated the enhancement of La incorporation and coating crystallinity. Microscopic observations further confirmed the formation of uniform and crack-free La-HA coatings composed of rod-like particles. These La-HA coatings showed a better degradation resistance in both acidic conditions and physiological ones, which prolong the survival of the coatings. The dope of La increased the bonding strength of coatings, and the highest values showed a resistance of up to 24.7 MPa, which was higher than mostly HA coatings with electrophoretic deposition or sol-gel methods prepared at room temperature. The La-HA coatings showed a positive trend toward cell proliferation and osteogenic differentiation when the content was below 20%. It is expected that the present La-HA coatings could be potentially used for Ti-based implants in the field of dental implantology. Further studies should be performed to identify their performance in modulating *in vitro* osteoblastogenesis and osteoclastogenesis as well as inan *in vivo* osteoporotic model.
